# *EARLY FLOWERING* is a dominant gain‐of‐function allele of *FANTASTIC FOUR 1/2c* that promotes early flowering in tomato

**DOI:** 10.1111/pbi.14217

**Published:** 2023-11-06

**Authors:** Dedi Zhang, Guo Ai, Kangna Ji, Rong Huang, Chunrui Chen, Zixuan Yang, Jiafa Wang, Long Cui, Guobin Li, Maryam Tahira, Xin Wang, Taotao Wang, Jie Ye, Zonglie Hong, Zhibiao Ye, Junhong Zhang

**Affiliations:** ^1^ National Key Laboratory for Germplasm Innovation & Utilization of Horticultural Crops Huazhong Agricultural University Wuhan China; ^2^ Hubei Hongshan Laboratory Wuhan China; ^3^ Department of Plant Sciences University of Idaho Moscow Idaho USA

**Keywords:** flowering time, *FANTASTIC FOUR*, *CSN5B*, *SFT*, tomato

## Abstract

Flowering time, an important factor in plant adaptability and genetic improvement, is regulated by various genes in tomato (*Solanum lycopersicum*). In this study, we characterized a tomato mutant, *EARLY FLOWERING* (*EF*), that developed flowers much earlier than its parental control. *EF* is a dominant gain‐of‐function allele with a T‐DNA inserted 139 bp downstream of the stop codon of *FANTASTIC FOUR 1/2c* (*FAF1/2c*). The transcript of *SlFAF1/2c* was at elevated levels in the *EF* mutant. Overexpressing *SlFAF1/2c* in tomato plants phenocopied the early flowering trait of the *EF* mutant. Knocking out *SlFAF1/2c* in the *EF* mutant reverted the early flowering phenotype of the mutant to the normal flowering time of the wild‐type tomato plants. *SlFAF1/2c* promoted the floral transition by shortening the vegetative phase rather than by reducing the number of leaves produced before the emergence of the first inflorescence. The COP9 signalosome subunit 5B (CSN5B) was shown to interact with FAF1/2c, and knocking out *CSN5B* led to an early flowering phenotype in tomato. Interestingly, FAF1/2c was found to reduce the accumulation of the CSN5B protein by reducing its protein stability. These findings imply that FAF1/2c regulates flowering time in tomato by reducing the accumulation and stability of CSN5B, which influences the expression of *SINGLE FLOWER TRUSS* (*SFT*), *JOINTLESS* (*J*) and *UNIFLORA* (*UF*). Thus, a new allele of *SlFAF1/2c* was discovered and found to regulate flowering time in tomato.

## Introduction

Flowering time is one of the most important agronomic traits because it influences the rate of vegetative growth and fruit yield (Soyk *et al*., 2017). Flowering indicates the transition from vegetative to reproductive growth in plants. The floral transition is initiated by a combination of factors that integrate endogenous genetic pathways with environmental stimuli (Andres and Coupland, 2012; Blumel *et al*., 2015; Eshed and Lippman, 2019). Various pathways that lead to flowering have been studied in *Arabidopsis* (Blumel *et al*., 2015). These pathways respond to both endogenous (autonomous, gibberellin, circadian rhythms and age) and environmental (vernalization, ambient temperature and photoperiod) cues (Albani and Coupland, 2010; Kim *et al*., 2009; Samach and Wigge, 2005; Wang, 2014). Recent studies on these pathways indicate that the mechanisms controlling the switch from vegetative to reproductive growth are complex (Blumel *et al*., 2015; Eshed and Lippman, 2019; Fornara *et al*., 2010; Meir *et al*., 2021).

The tomato is a sympodial plant with a scorpioid cymose inflorescence (Molinero‐Rosales *et al*., 1999). Through the acquisition of flowering mutants, traditional quantitative trait locus (QTL) mapping and QTL‐seq technology combined with modern high‐throughput sequencing, several classical genes involved in the control of flowering time in tomato have been cloned and characterized. *UNIFLORA* (*UF*), *COMPOUND INFLORESCENCE* (*S*), *SINGLE FLOWER TRUSS* (*SFT*), *JOINTLESS* (*J*) and *FALSIFLORA* (*FA*) exert their biological function to promote flowering, whereas *SELF‐PRUNING* (*SP*), *SELF PRUNING 5G* (*SP5G*) and *TERMINATING FLOWER* (*TMF*) have been shown to delay the flowering time in tomato.

Compared with *Arabidopsis*, which has been subject to intense genetic and molecular biology investigations on the flowering regulatory network, tomato is less well studied. There are two known main flowering regulatory pathways in tomato, including the photoperiodic pathway and the autonomous pathway (Dielen *et al*., 2004; Molinero‐Rosales *et al*., 2004). Tomato *UF*, *S* and *SP5G* genes play significant roles in photoperiod‐dependent flowering induction. Both *uf* and *s* mutants exhibit delayed flowering in winter (Dielen *et al*., 1998, 2001, 2004). Under low light conditions, the *s*/*uf* double mutant develops flowers significantly later than their corresponding single mutants (Quinet *et al*., 2006a). Genetic studies have shown that the *S* gene acts downstream of *UF* (Quinet *et al*., 2006a). *SP5G*, a homologue of *Arabidopsis FLOWERING LOCUS T* (*FT*), is induced by the long‐day photoperiod and plays a role in delaying flowering time in tomato (Soyk *et al*., 2017; Zhang *et al*., 2018b). Although modern tomatoes are day‐neutral plants and thus produce flowers regardless of the photoperiod length, the *sp5g* mutant flowers rapidly with less sensitivity to photoperiod (Soyk *et al*., 2017). *J* and *SFT* function in the autonomous pathway. Differing from its *Arabidopsis*, homologue *SHORT VEGETATIVE PHASE* (*SVP*), the tomato *J* gene has only a small role in flowering promotion (Hartmann *et al*., 2000; Szymkowiak and Irish, 2006). *SFT*, another tomato homologue of *Arabidopsis FT*, promotes early flowering and controls floral meristem identity and floral development (Molinero‐Rosales *et al*., 2004). In contrast to the wild‐type tomato, the *sft* mutant plants exhibit markedly delayed flowering times and, thus, the systemic SFT signals could function as the daytime flowering stimulus (Lifschitz *et al*., 2006; Molinero‐Rosales *et al*., 2004; Shalit *et al*., 2009). *SFT* has been considered as a possible flowering integrator of the photoperiodic pathway and the autonomous pathway, as it has been shown that the tomato flowering hormone florigen can substitute for the high light dose that is required for the *UF*‐regulated photoperiodic pathway (Lifschitz *et al*., 2006). Furthermore, *SFT* and *SP* are antagonistic in that the dosage of *SFT* influences the expression of *SP* and vice versa. This antagonism fine‐tunes shoot architecture and flowering time (Jiang *et al*., 2013; Krieger *et al*., 2010; Lifschitz *et al*., 2014). In addition, several genes, including *FA*, *TMF* and *BLADE ON PETIOLE* (*BOP*), involved in flowering regulatory pathways have not been well studied. Tomato *FA* is the orthologue of *Arabidopsis LAEFY* (*LFY*) (Molinero‐Rosales *et al*., 1999) and plays an important role in the control of floral meristem identity and in advancing flowering time (Thouet *et al*., 2012). The *sft/fa* double mutant does not flower, suggesting that *SFT* and *FA* act in two independent pathways to promote flowering time in tomato (Lozano *et al*., 2009; Molinero‐Rosales *et al*., 2004). *TMF* is a tomato ALOG family gene that inhibits flowering in tomato (MacAlister *et al*., 2012). Further studies showed that *TMF* interacts with three SlBOP proteins and recruits the BOP proteins to the nucleus to form a transcriptional complex. Similar to *tmf* mutant, the *bop1*/*2*/*3* triple mutant exhibits advanced flowering time in tomato (Xu *et al*., 2016).

The *FANTASTIC FOUR* (*FAF*) gene family was initially characterized in *Arabidopsis* (Wahl *et al*., 2010). The *Arabidopsis* genome encodes 10 FAF domain‐containing proteins, of which four belong to the FAF family and the remaining 6 are more diverse (Mu *et al*., 2017; Wahl *et al*., 2010). There are 13 FAF domain‐containing proteins in tomato, of which 5 are members of the FAF family (FAF1/2a, FAF1/2b, FAF1/2c, FAF3/4a and FAF3/4b), 4 are FAF‐like proteins and the remaining 4 are more diverse (Mu *et al*., 2017). It is interesting to note that *FAF* genes have not been found in the rice genome or any other monocotyledonous species, although proteins with similarities to the *Arabidopsis* FAF‐like proteins are present in these species (Wahl *et al*., 2010). No homologous proteins of FAF have been identified outside the plant kingdom, suggesting that the *FAF* gene family is plant‐specific or even eudicotyledonous‐specific (Wahl *et al*., 2010). In plants, studies on the function of the *FAF* gene family are limited. In *Arabidopsis*, the *FAF* gene family has been implicated in the regulation of shoot meristem size by modulating the CLAVATA 3 (CLV3)‐WUSCHEL (WUS) feedback loop (Wahl *et al*., 2010). Moreover, there has been evidence to show that the *FAF* gene family responds to photoperiodic changes (Torti *et al*., 2012; Wahl *et al*., 2010; You *et al*., 2019). In tomato, QTL *fw11.3* has been found to encode a *FAF* gene that promotes enlargement of the pericarp, increasing fruit weight (Mu *et al*., 2017).

In this study, we identified a T‐DNA insertion mutant, *EARLY FLOWERING* (*EF*), that developed flowers earlier than its parental line. *EF* was a dominant gain‐of‐function allele that led to the elevation of the *SlFAF1/2c* transcript. The *EF* mutant flowered early because the time from germination to the emergence of the first inflorescence was reduced. Transgenic tomato lines overexpressing *SlFAF1/2c* phenocopied the early flowering trait of the *EF* mutant. FAF1/2c interacted with photomorphogenic COP9 signalosome subunit 5B (CSN5B) and reduced its protein stability and accumulation. Transgenic lines with *CSN5B* knocked out also exhibited early flowering phenotypes. Our evidence further suggests that the FAF1/2c‐CSN5B module regulates flowering time by regulating the expression of *SFT*, *J* and *UF*. These results demonstrate a pivotal role of *FAF1/2c* in the regulation of flowering time and a new molecular mechanism underlying flowering time control in plants.

## Results

### *EF* is a dominant allele that promotes early flowering

**Figure 1:**
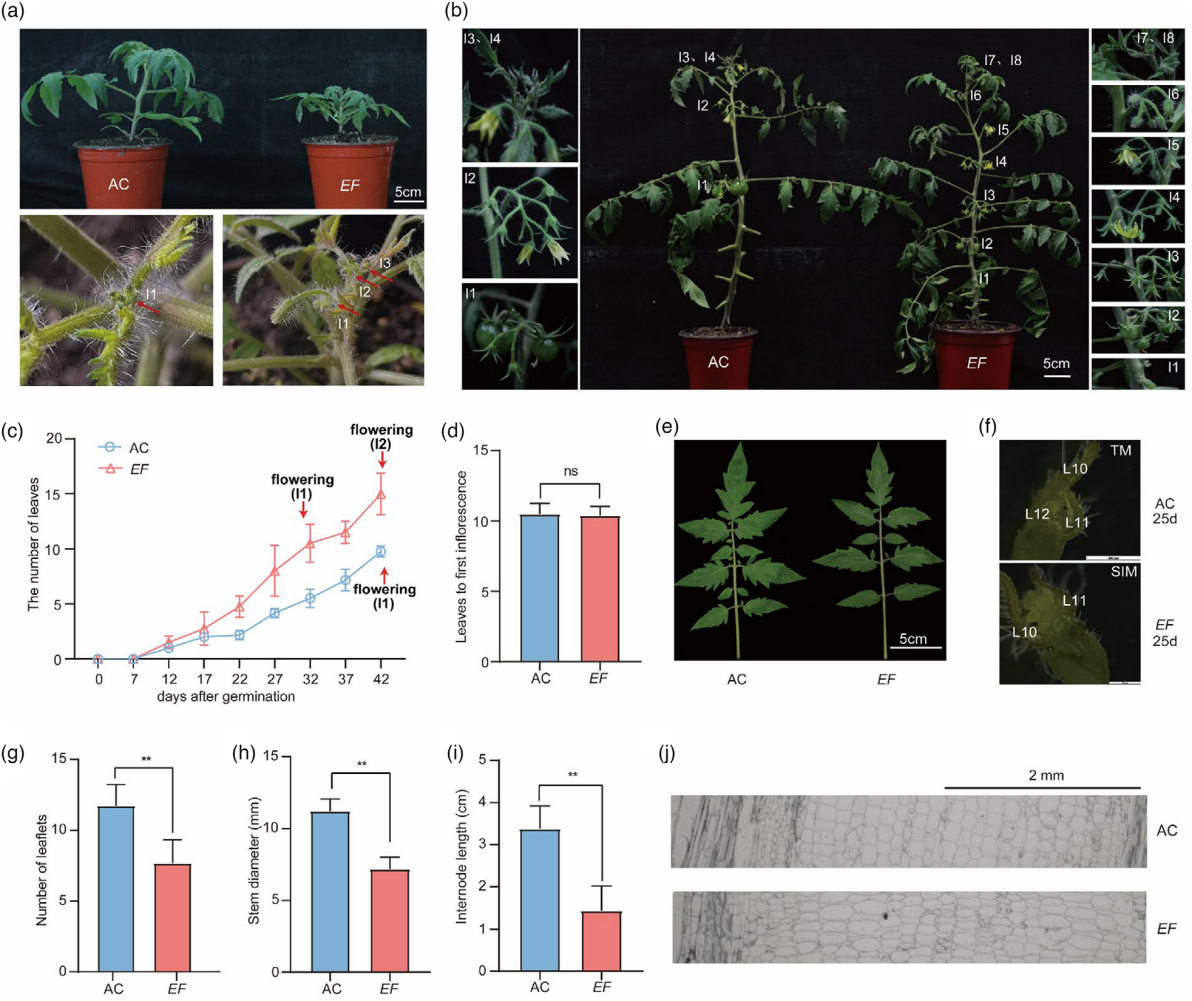
Phenotypic characterization of *EF* mutant plants. (a) Six‐week‐old wild‐type and *EF* mutant plants. *EF* mutant and wild‐type (AC) plants are shown (upper panel). Higher magnification images (lower panel) indicate fewer inflorescences in AC (left) than in *EF* mutants (right). Red arrows indicate the positions of inflorescences. I1, I2 and I3 represent the first, second and third inflorescences, respectively. Scale bar: 5 cm. (b) Two‐month‐old AC and *EF* mutant plants. I‐numbers indicate the positions of the inflorescences. Scale bar: 5 cm. (c) Flowering times of AC and *EF* grown in a greenhouse. Flowering time was measured by the number of days after seed germination and by the number of leaves below the first inflorescence. Red arrows indicate the emergence of the inflorescences. Flowering (I1) and Flowering (I2) represent the times of the emergence of the first and second inflorescences, respectively. *n* = 5. (d) The number of leaves below the first inflorescence in *EF* and AC. ns, not statistically significant. *n* = 5. (e) Mature leaves from AC and *EF*. Leaves from representative 6‐week‐old seedlings are shown. Scale bar: 5 cm. (f) Stereomicrographs of shoot apical meristems from the primary shoots from AC and *EF*. Shoot apical meristems from 25‐day‐old plants were analysed.TM, transition meristem; SIM, sympodial inflorescence meristem. Leaf (L) numbers are as marked. Scale bar: 500 μm. Numbers of leaflets (g), stem diameters (h) and internode lengths (i) in 6‐week‐old AC and *EF* seedlings. Stem diameters and internode lengths were measured between the second and third truss. **, *P* < 0.01 (*t*‐test). (j) Longitudinal sections of the stems between the second and third truss from AC and *EF*.

We identified a T‐DNA insertion mutant (*EF*) with an abnormal flowering time (Figure 1a–d,f), height (Figure 1a), stem diameter (Figure 1h), leaf shape (Figure 1e,g) and internode length (Figure 1i,j). The *EF* mutants were significantly shorter (Figure 1a), produced fewer leaflets in their mature leaves (Figure 1e,g), produced stems with significantly smaller diameters (Figure 1h) and had markedly reduced internode length (Figure 1i,j) relative to the AC plants. Most noticeably, the *EF* mutant flowered early (Figure 1c), with a shorter time between the development of successive flowers (Figures 1b and S1a) and a reduction in fruit weight (Figure S1b). The height difference between *EF* mutant plants and AC plants decreased gradually as the plants developed (Figures 1a,b and S1a). In this study, we characterized the early flowering time phenotype of *EF* because this was perhaps the most striking phenotype of the mutant and because the early‐flowering phenotypes would be potentially useful for the tomato industry. We quantified the flowering time by measuring the number of days from seed germination to the emergence of the first inflorescence and also by recording the number of leaves below the first inflorescence. Although the number of leaves below the first inflorescence was not significantly different between *EF* and AC (Figure 1c,d), the number of days from germination to the development of the first flower was significantly less in *EF* than in AC (Figure 1c). Both AC and *EF* produced approximately 10 real leaves before the development of the first inflorescence. However, while AC produced its first inflorescence at 42 days, the *EF* mutant required only 32 days to produce its first inflorescence. We found that after 25 days, the shoot apical meristems of the *EF* mutant had developed to the sympodial inflorescence meristem (SIM) stage and the shoot apical meristem of AC remained at the transition meristem (TM) stage (Figure 1f).

The *EF* mutant was crossed to AC, and all the plants in the F_1_ generation were phenotypically similar to *EF* in that they flowered early, suggesting that the early flowering trait in *EF* was dominant over the normal flowering of AC. Of 100 F_2_ plants, 71 were early flowering and 29 were “normal” flowering, which fit the Mendelian segregation ratio of 3:1 (*χ* ^2^ = 0.65, *P* > 0.05). From the F_2_ population, we randomly selected 47 plants for PCR analysis of genomic DNA. We found that 16 plants were homozygous (+/+) and 18 plants were hemizygous (+/−) for T‐DNA insertion. The remaining 13 plants did not contain T‐DNA insertion (−/−). Both the homozygous (+/+) and heterozygous (+/−) plants flowered early and were phenotypically similar to the *EF* mutant. In contrast, the plants lacking T‐DNA insertion flowered at the same time as AC. Thus, the *EF* mutation is a dominant allele.

### *EF* encodes *SlFAF1/2c*, a member of the *FAF* gene family

**Figure 2:**
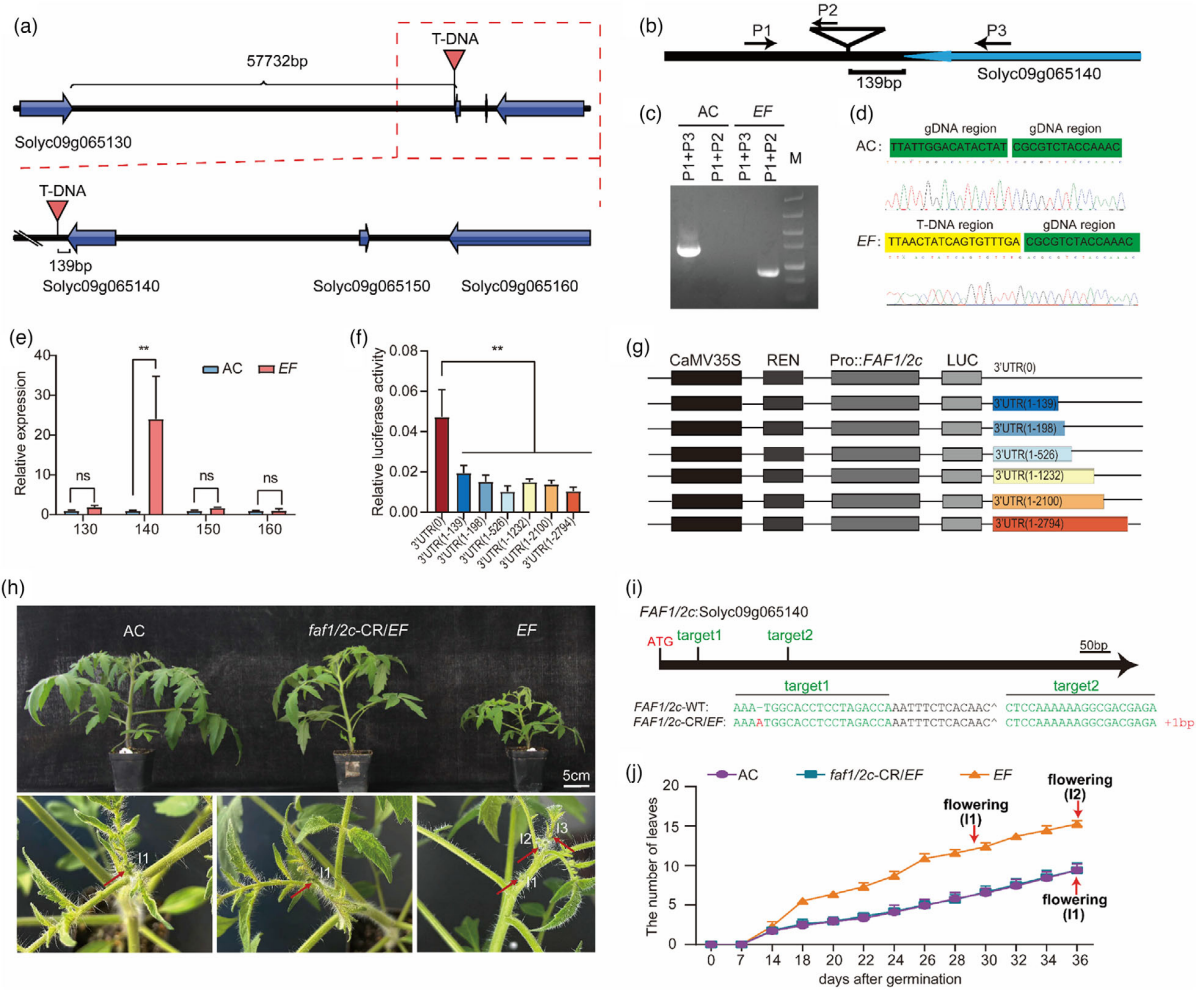
Elevated expression of *SlFAF1/2c* in the *EF* mutant. (a) Diagram of the genomic region flanking the T‐DNA insertion. The insertion site was determined using TAIL‐PCR. The black line represents the genomic DNA. The thick blue arrows represent annotated genes and the direction of transcription on chromosome 9. The red arrowhead indicates the T‐DNA insertion site. (b) Schematic diagram indicating the locations of the primers used for determination of the location of the T‐DNA insertion site. P1, P2 and P3 represent primer 1, primer 2 and primer 3, respectively. (c) PCR products were generated using different combinations of primers and genomic DNA from AC and *EF*. (d) Sequence analysis of the T‐DNA insertion site in the *EF* mutant. The sequences of PCR products from (c) in AC (above) and the *EF* mutant (below). (e) Relative transcript levels of genes flanking the T‐DNA insertion site. Relative expression was quantified using qRT‐PCR. Solyc09g065130, Solyc09g065140, Solyc09g065150 and Solyc09g065160 are represented by 130–160, respectively. (f, g) Relative luciferase activity was measured as the ratio of the firefly luciferase (LUC) activity to the *Nenilla* luciferase (REN) activity. The CaMV35S promoter was used to drive the expression of REN. The native promoter (3 kb) from *SlFAF1/2c* was used to express LUC. Different lengths of the 3′‐UTR from *SlFAF1/2c*, as indicated with nucleotide numbers starting from the translation stop codon, were fused to the LUC coding sequence. All data are shown as mean values ± SE (*n* = 5). **, *P* < 0.01 (*t*‐test). (h) Six‐week‐old AC, *faf1/2c*‐CR/*EF* and *EF* seedlings. Scale bar: 5 cm. High‐magnification images of shoot apices from AC, *faf1/2c*‐CR/*EF* and *EF*, respectively. Red arrows mark the inflorescences. I1, I2 and I3 represent the first, second and third inflorescences, respectively. (i) A schematic diagram of the genomic DNA from *FAF1/2c* showing the binding sites of the two guide RNAs (target 1 and target 2) used to induce a mutation in the *EF* mutant background using CRISPR/Cas9 technology. The *FAF1/2c* gene contains only one exon. The locations of target 1, target 2 and the translation start codon ATG are indicated. The genomic DNA sequences from *FAF1/2c* with the 1‐bp insertion in *faf1/2c*‐CR/*EF* are compared with AC. The 1‐bp insertion in the target region is a frame‐shift mutation in the *FAF1/2c* coding region in the *faf1/2c*‐CR/*EF* line. (j) Comparison of flowering times in AC, *EF* and the *faf1/2c*‐CR/*EF* double mutant plants grown in a greenhouse. *n* = 5.

To identify the location of the T‐DNA insertion in the genome of *EF* mutants, we used TAIL‐PCR to amplify the genomic DNA fragments adjacent to the border of the T‐DNA. The DNA sequences amplified from the *EF* mutant were used as queries to search the Sol Genomics Network database. We found that the T‐DNA was inserted into the 3′‐untranslated region (UTR) of Solyc09g065140 on chromosome 9 (Figure 2a,b). This insertion was located only 139 bp downstream of the stop codon of Solyc09g065140 and was 57 593 bp downstream of the stop codon of Solyc09g065130 (Figure 2a). The T‐DNA insertion site in the genome was independently confirmed by amplifying the insertion site with specific primers (Figure 2b–d). To test whether the T‐DNA insertion affected the expression of genes near the insertion site, we quantified the relative expression levels of Solyc09g065130, Solyc09g065140, Solyc09g065150, and Solyc09g065160 in leaves (Figure 2e) and found that the expression level was increased significantly only in Solyc09g065140, whereas the expression of the three other genes remained unchanged. To further study the function of the 3′‐UTR, a dual luciferase reporter assay was conducted. Fragments of different lengths from the 3′‐UTR were fused with the coding sequence of firefly luciferase driven by the native promoter of Solyc09g065140 (Figure 2f,g). The results showed that the 3′‐UTR reduced the expression levels of the firefly luciferase. The cause of this reduction in gene expression remained unknown. It is possible that the 3′‐UTR of Solyc09g065140 may contain a *cis‐*regulatory element (CRE) that represses the promoter activity of Solyc09g065140. It is also possible that the 3′‐UTR may destabilize the mRNA, resulting in a reduction in its transcript level. In either of the two cases, a T‐DNA insertion would disrupt the 3′‐UTR and cause an elevation in the transcript level of Solyc09g065140. Taken together, these findings indicate that the T‐DNA insertion in the 3′‐UTR of Solyc09g065140 serves as a gain‐of‐function mutation that blocks the function of the 3′‐UTR and thus, leads to elevated expression of Solyc09g065140.

Solyc09g065140 encodes *SlFAF1/2c*, which is a member of the *Fantastic Four* (*FAF*) gene family (Mu *et al*., 2017). Sequence analysis shows that *SlFAF1/2c* has an uninterrupted open reading frame (ORF) of 732 bp without any intron and encodes a polypeptide of 203 amino acid residues with a conserved FAF domain between amino acid residues 139 and 191 (Figure S2a). The SlFAF1/2c protein shares 35.0%, 30.8%, 32.6% and 37.8% amino acid sequence identity with FAF1, FAF2, FAF3 and FAF4 from *Arabidopsis*, respectively (Figure S2b). *SlFAF1/2c* was expressed at very different levels in different tissues in AC plants. The transcript levels were highest in leaves and roots, especially in young leaves, and were relatively low in flowers and nearly undetectable in fruits at different developmental stages (Figure S2c).

To test if the increased *SlFAF1/2c* expression was the cause for the early flowering phenotype in the *EF* mutant, we used CRISPR technology to knock out the *FAF1/2c* coding region (*faf1/2c*‐CR) in the *EF* mutant background and thus generated an allele of *FAF1/2c* containing two mutations (*faf1/2c*‐CR/*EF*) (Figure 2h,i). The double mutant flowered later than the *EF* mutant and at the same time as the AC control (Figure 2h,j). These data are consistent with the hypothesis that the enhanced expression levels of a functional *SlFAF1/2c* gene were the cause for the early flowering phenotype of the *EF* mutant and clearly show that the T‐DNA insertion in *EF* is a gain‐of‐function mutation that was associated with the elevated expression of *FAF1/2c* and early flowering in the mutant.

### Overexpression of *FAF1/2c* leads to early flowering

**Figure 3:**
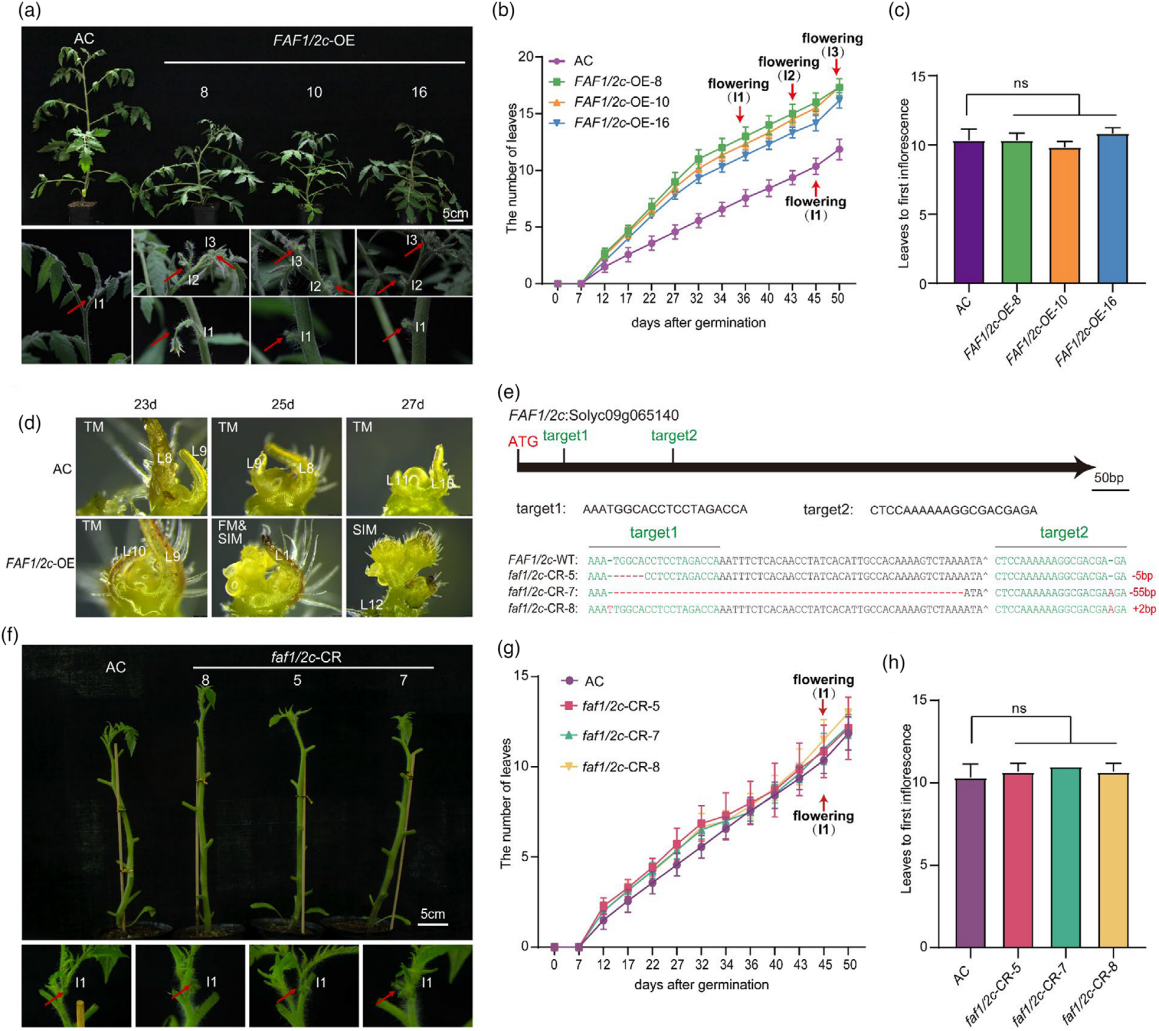
Early flowering in *FAF1/2c* overexpressing lines. (a) Six‐week‐old AC and *FAF1/2c*‐OE seedlings. Wild‐type (AC) and *FAF1/2c*‐OE lines 8, 10 and 16 are shown. Scale bar: 5 cm. Magnification of shoot apices from AC and the *FAF1/2c*‐OE lines. Red arrows indicate the positions of inflorescences. I1, I2 and I3 indicate the first, second and third inflorescences, respectively. More inflorescences were produced in *FAF1/2c*‐OE lines than in AC plants. (b) Flowering time of the *FAF1/2c*‐OE lines (OE‐8, ‐10 and ‐16) relative to AC. Red arrows indicate the emergence of inflorescences. Flowering (I1), Flowering (I2) and Flowering (I3) represent the times of the emergence of the first, second and third inflorescence, respectively. (c) Number of leaves below the first inflorescence in the AC and *FAF1/2c*‐OE lines. ns, no statistically significant difference (*t*‐test). *n* = 6. (d) Stereomicrographs of the primary shoot apical meristems from AC and the *FAF1/2c*‐OE lines. Plants at 23 (left), 25 (middle) and 27 (right) days after germination. TM, transition meristem; SIM, sympodial inflorescence meristem. Leaf (L) numbers are as marked. Scale bar: 100 μm. (e) Diagram of the genomic DNA of *FAF1/2c*. The locations of the two single‐guide RNAs (target 1 and target 2) used to create the CRISPR/Cas9‐engineered mutations are indicated. The locations of target 1, target 2 and the translation start codon (ATG) are indicated. The sequences of the two sgRNAs are shown below the *FAF1/2c* gene structure. The genomic DNA sequence of *FAF1/2c* from wild‐type (AC) containing the two target sequences is shown. The genomic DNA sequences from CR‐5, CR‐7 and CR‐8 are also shown. *faf1/2c*‐CR‐5 has a 5‐bp deletion. *faf1/2c*‐CR‐7 has a 55‐bp deletion and a 1‐bp insertion. *faf1/2c*‐CR‐8 has two 1‐bp insertions. (f) Comparison of the primary shoots from the *faf1/2c*‐CR lines (CR‐5, CR‐7 and CR‐8) and AC. Leaves were detached before photographing. Images of the apices are shown below their primary shoots. Red arrows indicate the positions of inflorescences. Scale bar: 5 cm. (g) Flowering time of *faf1/2c*‐CR lines (CR‐5, CR‐7 and CR‐8) relative to AC. Red arrows indicate the emergence of the first inflorescence. Mean values ± SE are shown. (h) Number of leaves below the first inflorescence in AC and the *faf1/2c*‐CR lines. ns, no statistically significant difference was determined with a *t*‐test. *n* = 6.

To obtain further evidence that *FAF1/2c* was indeed the causal locus for the early flowering phenotype of the *EF* mutant, we generated transgenic lines overexpressing *FAF1/2c* in the AC background (*FAF1/2c*‐OE in AC) (Figure S3a) and loss‐of‐function mutations in *FAF1/2c* in the AC background (*faf1/2c*‐CR in AC) using CRISPR/Cas9 technology (Figure 3e). Increases in the expression levels of *SlFAF1/2c* led to a drastic reduction in the flowering time in the *FAF1/2c*‐OE lines relative to AC plants (Figure 3a–d), consistent with the early flowering phenotype of the *EF* mutant. In addition, as compared with the AC plants, the phenotypes such as simpler leaves and shorter internode length in the *FAF1/2c*‐OE lines were similar to those of the *EF* mutant plants (Figure S3b,c). Stereomicroscopic analysis of the primary shoot apical meristems from AC and the *FAF1/2c*‐OE lines at 23 days, 25 days and 27 days post‐germination indicated an earlier transition to the inflorescence meristem in *FAF1/2c*‐OE lines relative to AC plants (Figure 3d). The *FAF1/2c*‐OE lines produced the first inflorescence approximately 35 days after germination. However, the *faf1/2c*‐CR lines developed the first inflorescence at approximately 45 days after germination, which was not significantly different from AC plants (Figure 3f–h). These data imply that *FAF1/2c* is not essential for maintaining the ‘normal’ or default flowering time in tomato. However, an increased expression level of *FAF1/2c* promotes early flowering regardless of whether the elevated expression comes from the overexpression of *FAF1/2c* in the *FAF1/2c*‐OE lines or the T‐DNA insertion in the 3′‐UTR of *SlFAF1/2c* in the *EF* mutant. Therefore, *FAF1/2c* is a new key gene for inducing flowering time in tomato.

### FAF1/2c interacts with CSN5B *in vitro* and *in vivo*

**Figure 4:**
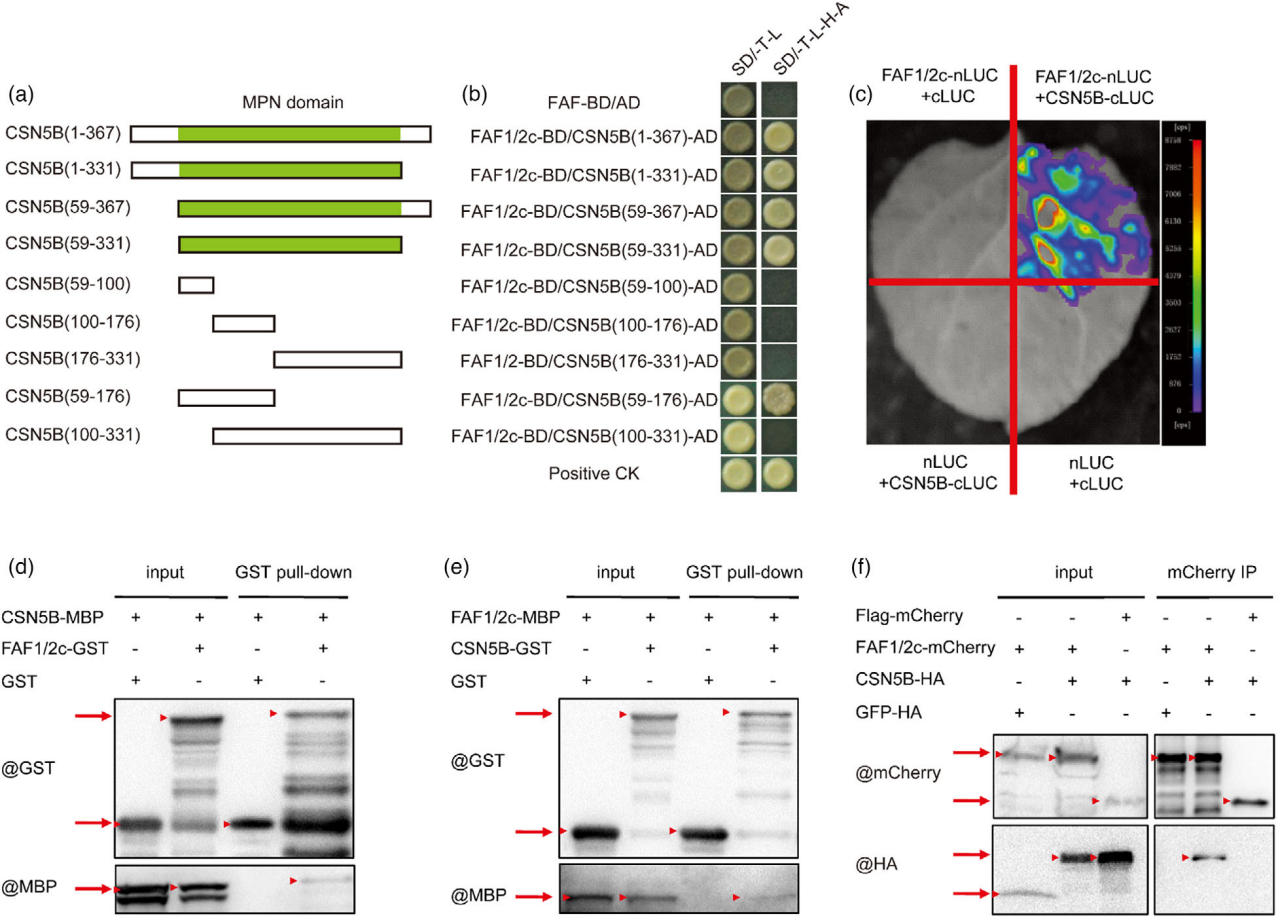
Protein–protein interactions between FAF1/2c and CSN5B. (a) Schematic diagram of the CSN5B protein and CSN5B protein fragments. Amino acid residue numbers are indicated in parentheses. The Mpr1 and Pad1 N‐terminus (MPN) domains (Jin *et al*., 2014) are shown in green. (b) Yeast two‐hybrid assays. The CSN5B protein and CSN5B protein fragments were fused to the activation domain (AD) of GAL4. FAF1/2c was fused to the DNA‐binding domain (BD) of GAL4. A strain containing pGADT7‐T and pGBKT7‐53 (Clontech) was used as a positive control (positive CK). (c) Split‐luciferase assays. FAF1/2c and CSN5B were fused with either the N‐terminal (nLUC) or C‐terminal (cLUC) portion of firefly luciferase (LUC). *N. benthamiana* leaves were infiltrated with *Agrobacterium* cells containing different combinations of constructs. Chemiluminescence was imaged after incubation with the substrate, luciferin. (d, e) Pull‐down assays. FAF1/2c and CSN5B were fused with the GST tag and MBP tag, respectively (d). In a parallel experiment, FAF1/2c and CSN5B were fused with the MBP tag and the GST tag, respectively (e). The recombinant proteins were expressed in *E. coli* cells, purified and immunoprecipitated. After incubation, the protein mixture was passed through a glutathione‐Super‐flow resin and detected using anti‐GST and anti‐MBP antibodies, respectively. Red arrows from top to bottom indicate the positions of the expected bands of FAF1/2c‐GST, GST and CSN5B‐MBP, respectively (d). Red arrows from top to bottom indicate the positions of the expected bands of CSN5B‐GST, GST and FAF1/2c‐MBP, respectively (e). (f) Co‐IP assays. FAF1/2c and CSN5B were fused with the mCherry tag and the HA tag, respectively. *N. benthamiana* leaves were infiltrated with *Agrobacterium* cells containing different combinations of constructs. Proteins were immunoprecipitated (IP) with anti‐mCherry antibodies and detected with anti‐mCherry and anti‐HA antibodies. Flag‐mCherry and GFP‐HA served as control proteins. Red arrows from top to bottom indicate the positions of expected bands of FAF1/2c‐mCherry, Flag‐mCherry, CSN5B‐HA and GFP‐HA, respectively. Red triangles indicate the positions of the expected bands.

To gain insight into the mechanism influenced by *FAF1/2c*, we performed a yeast two‐hybrid screen for proteins that might interact with FAF1/2c. COP9 signalosome (CSN) subunit 5B (CSN5B) was identified among other proteins as an interactor of FAF1/2c and was of great interest to us because its role in flowering time control has not been reported, although CSN5B has been implicated in photomorphogenesis in higher plants (Jin *et al*., 2014; Wei *et al*., 2008). We first tested different domains of the CSN5B protein and found that the N‐terminus (59–176 aa) of the Mpr1 and Pad1 N‐terminus (MPN) domains of CSN5B interacted with FAF1/2c (Figure 4a,b). FAF1/2c and CSN5B also interacted in split‐luciferase (Figure 4c) and co‐immunoprecipitation (Co‐IP) assays (Figure 4f), indicating that a FAF1/2c‐CSN5B protein complex formed *N. benthamiana* leaves. *In vitro*, pull‐down assays showed that recombinant FAF1/2c protein interacted with recombinant CSN5B protein with different tags (Figure 4d,e). Taken together, these results demonstrate that the interactions between FAF1/2c and CSN5B occurred both *in vitro* and *in vivo*.

### *CSN5B* plays a role in the regulation of flowering time in tomato

**Figure 5:**
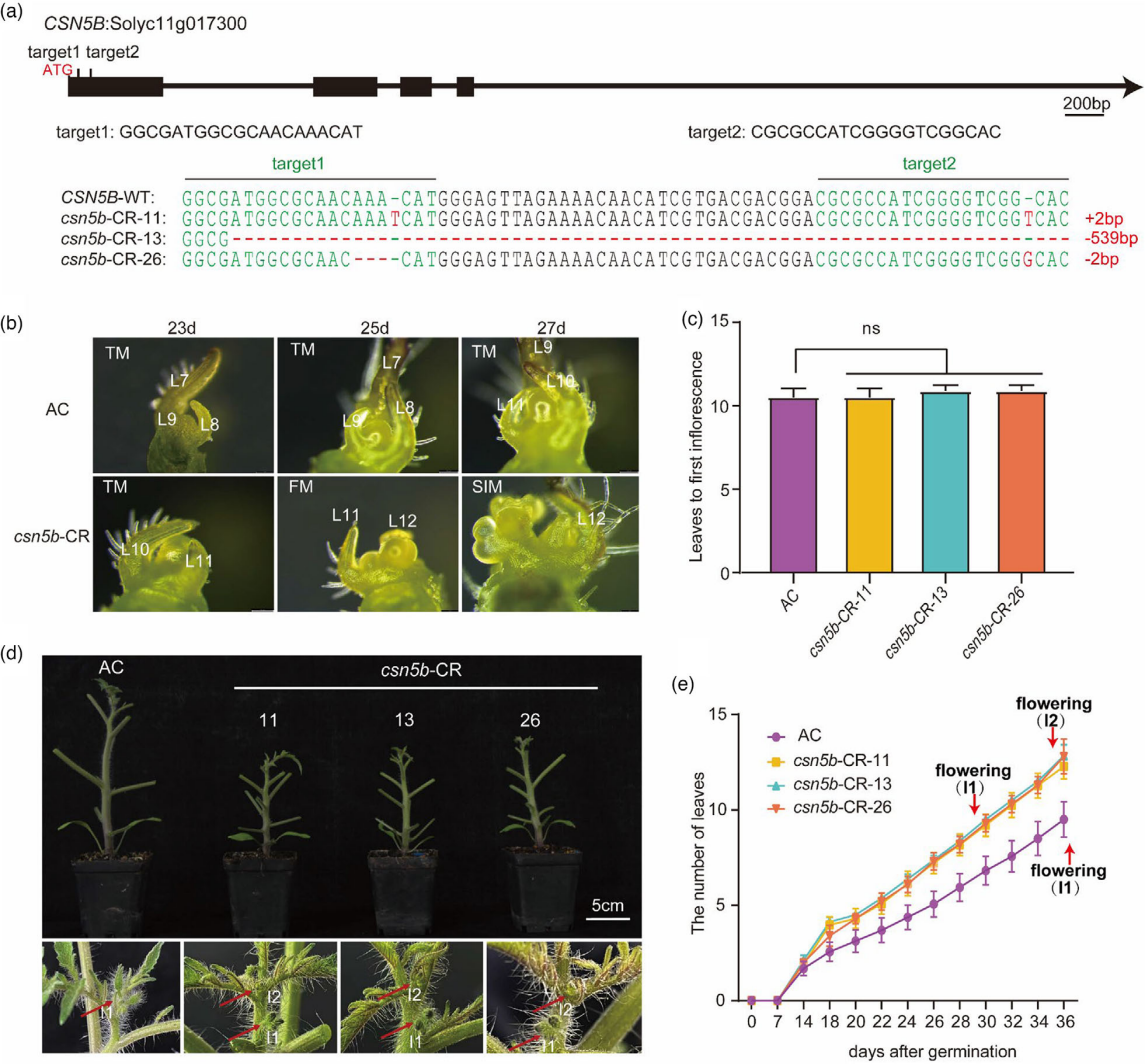
Early flowering phenotype of *CSN5B* knockout lines. (a) Diagram of the *CSN5B* gene and sequences of *CSN5B* null alleles. Black boxes, black lines and black arrows represent exons, introns and the direction of transcription, respectively. The locations of the translation start codon (ATG) and the two single‐guide RNAs (sgRNAs, target 1 and target 2) in exon 1 are indicated. The sequences of the two sgRNAs are shown below in the *CSN5B* gene diagram. The genomic DNA sequences of the *CSN5B* alleles with the two target sequences highlighted in green are shown. There is a 2‐bp insertion in *csn5b*‐CR‐11, a deletion of 539 bp in *csn5b*‐CR‐13 and a 2‐bp deletion in *csn5b*‐CR‐26. (b) Stereomicrographs of the primary shoot apical meristems from AC and *CSN5B* knockout lines. Plants at 23 (left), 25 (middle) and 27 (right) days after germination. TM, transition meristem; FM, floral meristem; SIM, sympodial inflorescence meristem. Leaf (L) numbers are indicated. Scale bar: 100 μm. (c) Number of leaves below the first inflorescence in AC and *CSN5B* knockout lines. ns, no statistically significant difference as determined with a *t*‐test, *n* = 8. (d) Six‐week‐old AC and *csn5b*‐CR (CR‐11, CR‐13, CR‐26) seedlings. Leaves were detached before photographing. Images of the apices are shown below their primary shoots. Scale bar: 5 cm. High‐magnification images of shoot apices from AC and *csn5b*‐CR lines. Red arrows indicate the inflorescences. I1 and I2 represent the first and second inflorescences, respectively. (e) Flowering time in *csn5b*‐CR and AC based on leaf number below the first inflorescence. Red arrows indicate the times of the emergence of the inflorescence. Flowering (I1) and Flowering (I2) represent the times of the emergence of the first and second inflorescence, respectively. All the data shown are mean values ± SE.

To test whether CSN5B regulates flowering time by interacting with FAF1/2c, we generated transgenic plants that either overexpressed *CSN5B* (Figure S4) or expressed knockout alleles of *CSN5B* in the AC background (Figure 5a). For the phenotype analysis of the *CSN5B* knockout plants, we selected three mutant lines (*csn5b*‐CR) with disrupted reading frames. There was an insertion of 2 bp in *csn5b*‐CR‐11, a long deletion of 539 bp in *csn5b*‐CR‐13 and a 2‐bp deletion in *csn5b*‐CR‐26 (Figure 5a). The phenotypes of the *csn5b*‐CR knockout mutant plants were dramatic in that they produced inflorescences approximately 6–10 days earlier than the AC plants (Figure 5b–e). This early flowering phenotype was similar to the early flowering phenotype of the *EF* mutant (Figure 1) and the *FAF1/2c*‐OE plants (Figure 3). In contrast, the flowering times of the *CSN5B*‐OE lines were not significantly different from the flowering times of the AC control (Figure S4). These observations indicate that *CSN5B* regulates flowering time in tomato and may play a role in delaying flowering time in tomato because its knockout was found to lead to early flowering in *csn5b*‐CR lines.

### Role of FAF1/2c on protein stability of CSN5B

The results in Figures 3a and 5d clearly showed that overexpression of *FAF1/2c* and knockout of *csn5b* both led to the early flowering phenotypes in tomato. The opposite effects of *FAF1/2c* and *CSN5B* on flowering time prompted us to speculate that they might have an antagonistic effect at the protein level.

**Figure 6:**
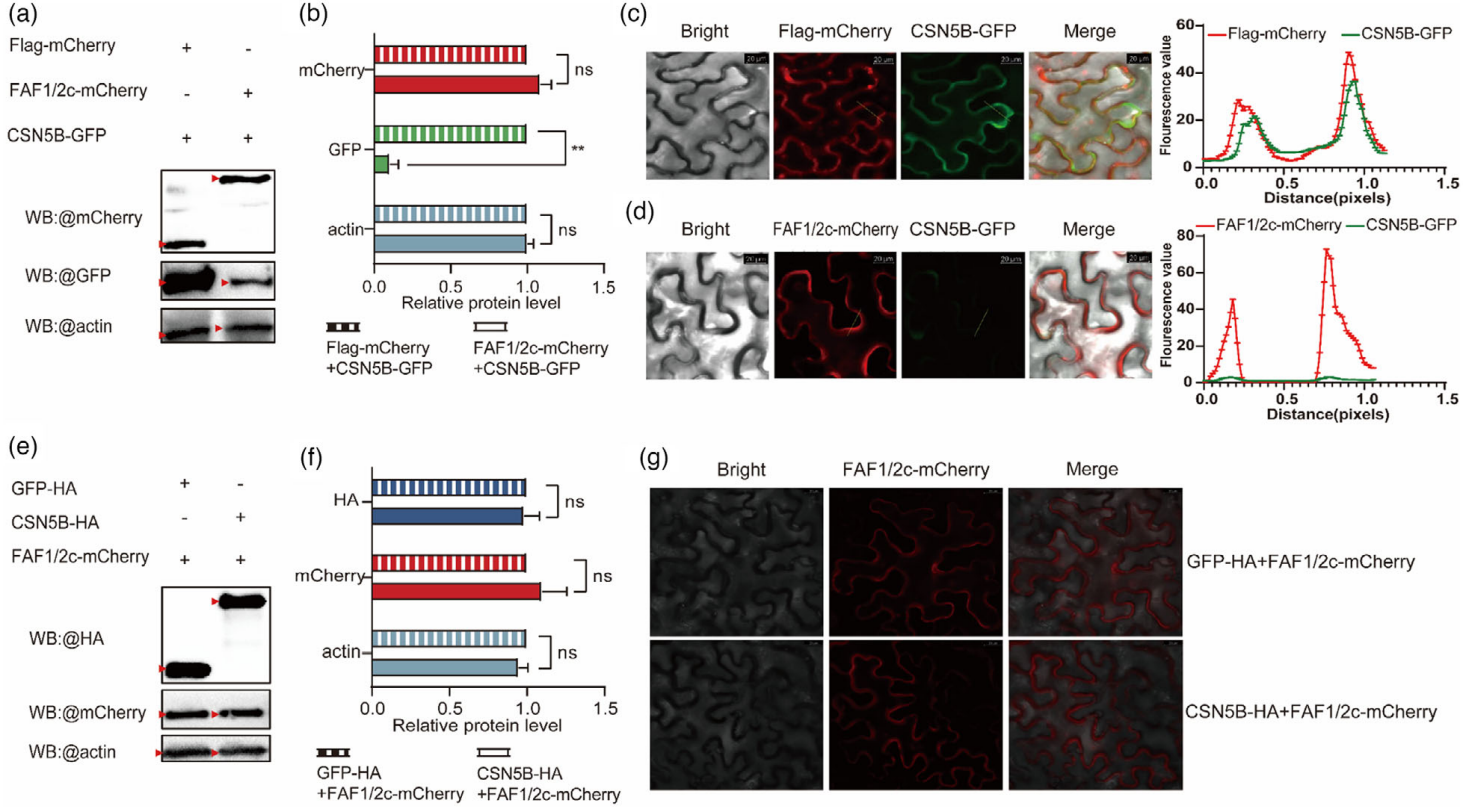
Interaction of FAF1/2c with CSN5B reduces the protein stability of CSN5B. (a) Western blot assays. FAF1/2c and CSN5B were co‐expressed as fusion proteins with the mCherry tag and GFP tag, respectively (right lane). Flag‐mCherry and CSN5B‐GFP were co‐expressed in the control group (left lane). Recombinant proteins were expressed in *N. benthamiana* leaves using *Agrobacterium* infiltration and detected on Western blots using anti‐mCherry, anti‐GFP and anti‐actin antibodies. Red triangles indicate the positions of the expected bands. (b) Quantification of the relative protein intensity in a. The protein level of the control group (Flag‐mCherry + CSN5B‐GFP) was set at 1.0. Three independent experiments were performed. **, *P* < 0.01. (c) Fluorescence images of *N. benthamiana* leaf cells co‐expressing Flag‐mCherry and CSN5B‐GFP. The fluorescence intensity of the indicated white dashed lines was quantified (right). Scale bars: 200 μm. (d) Fluorescence images of *N. benthamiana* leaf cells co‐expressing FAF1/2c‐mCherry and CSN5B‐GFP. The fluorescence intensity of the dashed lines was quantified (right). Scale bars: 200 μm. (e) Western blot assays. Western blots were performed using mCherry, HA and actin antibodies, respectively. Red triangles indicate the positions of the expected protein bands. (f) Quantification of the relative protein levels in e. The protein level of the control group (GFP‐HA + FAF1/2c‐mCherry) was set at 1.0. Three independent experiments were performed. (g) Fluorescence micrographs of FAF1/2c‐mCherry co‐expressed with GFP‐HA and CSN5B‐HA. Scale bars: 200 μm.

To test this hypothesis, we first tested whether FAF1/2c affected the stability of the CSN5B protein. We transiently co‐expressed FAF1/2c‐mCherry with CSN5B‐GFP as the test group, and Flag‐mCherry with CSN5B‐GFP as the control group in different sites of the same *N. benthamiana* leaf. Immunoblotting analysis demonstrated that co‐expression of CSN5B‐GFP with FAF1/2c‐mCherry led to a clear decrease in the levels of the CSN5B‐GFP protein (Figure 6a,b). Flag‐mCherry did not affect the stability of the CSN5B‐GFP protein (Figure 6a,b). Fluorescence images provided further evidence to support this interpretation (Figure 6c,d). When FAF1/2c‐mCherry and CSN5B‐GFP were co‐expressed in the same cell, stronger red fluorescence from the membrane was accompanied by weaker green fluorescence (Figure 6c). However, co‐expressing Flag‐mCherry and CSN5B‐GFP did not affect CSN5B‐GFP green fluorescence intensity (Figure 6d). Together, our immunoblotting analysis and fluorescence imaging provided compelling evidence that FAF1/2c reduced the protein stability of CSN5B. We also investigated whether CSN5B affected the stability of the FAF1/2c protein by Western blotting (Figure 6e,f) and fluorescence microscopy (Figure 6g) and found that CSN5B did not affect the protein stability of FAF1/2c.

Based on the above findings, we conclude that *FAF1/2c* regulates flowering time in tomato by interacting with CSN5B, resulting in a decrease in the protein stability of CSN5B.

### *SlFAF1/2c* promotes flowering by up‐regulating the expression of *SFT*, *J* and *UF*

**Figure 7:**
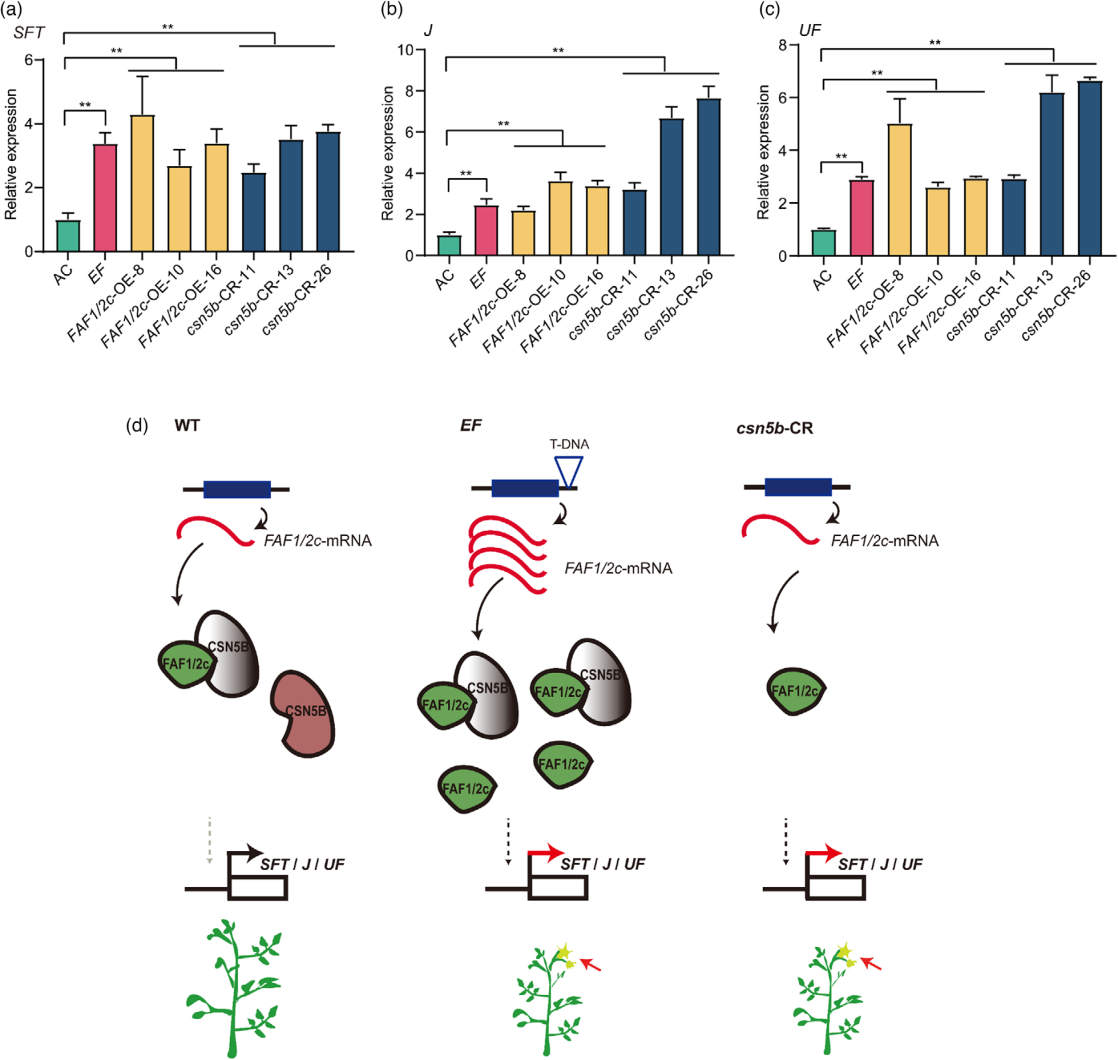
Model for the regulation of flowering time by the FAF1/2c‐CSN5B module in tomato. (a–c) Effect of *FAF1/2c* and *CSN5B* on the expression of *SFT*, *J* and *UF*. Relative expression levels of *SFT* (a), *J* (b) and *UF* (c) in the *EF* mutant, *FAF1/2c*‐OE and *csn5b*‐CR lines. The expression levels of *SFT*, *J* and *UF* in the control AC plants were set at 1.0. **, *P* < 0.01, *t*‐test, (*n* = 3). (d) Model for the determination of flowering time by FAF1/2c and CSN5B. In this model, FAF1/2c and CSN5B are positive and negative regulators of *SFT*, *J* and *UF* gene expression, respectively. The interaction of the two proteins leads to degradation of CSN5B. In wild‐type plants, the protein level of FAF1/2c is much lower than that of CSN5B, and their interaction could only partially down‐regulate the protein level of CSN5B. The remaining CSN5B proteins are sufficient to control the ‘normal’ flowering time. In *EF* mutant plants, the T‐DNA insertion in the 3′‐UTR of *FAF1/2c* leads to the accumulation of *FAF1/2c* transcript and FAF1/2c protein. The increased FAF1/2c level in *EF* plants could completely overcome the negative effect of CSN5B and promote early flowering. In *csn5b*‐CR lines, CSN5B is not produced, and the ‘normal’ level of FAF1/2c proteins is sufficient to promote early flowering by increasing *SFT*, *J* and *UF* gene expression. Thus, a fine balance between FAF1/2c and CSN5B proteins plays a critical role in the regulation of flowering time in tomato. Inactive CSN5B proteins due to interaction with FAF1/2c are shown in grey, while the remaining free and active CSN5B proteins are indicated in dark red.

We selected a group of genes that have been implicated in flowering time control in tomato and measured their expression levels in the *EF* mutant, *FAF1/2c*‐OE lines and *csn5b*‐CR lines using qRT‐PCR. We found that the expression of the *SFT* (Solyc03g063100), *J* (Solyc11g010570) and *UF* (Solyc09g005070) genes were up‐regulated significantly in the *EF* mutant, *FAF1/2c*‐OE lines and *csn5b*‐CR lines (Figure 7a–c). The expression of the *FA* (Solyc03g118160), *S* (Solyc02g077390), *SP* (Solyc06g074350), *TMF* (Solyc09g090180) and *BOP* (Solyc04g040220) genes did not change significantly and remained within the range of a two‐fold difference between the AC plants and the *EF* mutant, *FAF1/2c*‐OE lines and *csn5b*‐CR lines, respectively (Figure S5b–f). These results imply that *FAF1/2c* promotes flowering by up‐regulating the expression of flower‐promoting genes such as *SFT*, *J* and *UF* in tomato. The expression of *SP5G* (Solyc05g053850) was not significantly different among the *EF* mutant, the *FAF1/2c*‐OE lines and the AC control, but was significantly down‐regulated in the *csn5b*‐CR lines (Figure S5a). These data provided evidence that the expression of the key flowering regulatory gene *SP5G* may be regulated by *CSN5B*.

## Discussion

In this work, we cloned and characterized an *EF* allele that promotes early flowering in tomato by inducing the expression of *FAF1/2c*. Our results demonstrate that *EF* is a dominant gain‐of‐function allele of *FAF1/2c* (Figures 1 and 2) and a novel regulator of flowering time in tomato. *FAF1/2c*, a member of the *FAF* gene family (Wahl *et al*., 2010), encodes a protein with the unique FAF domain (Pfam accession PF11250). Previous studies have shown that *FAF* genes control the size of shoot meristems and that enhancing the expression of *FAF* genes reduces the size of the shoot apical meristem and leaf vasculature in *Arabidopsis* (Wahl *et al*., 2010). There have been no previous reports, to our knowledge, that indicate the role of *FAF* genes in flowering time control in plants. We speculate that the low sequence similarity between the tomato and *Arabidopsis* FAF proteins (Figure S5b) may have resulted from sequence divergence during the process of evolution and is consistent with FAF proteins evolving different functions in different plant species. In this study, the increased expression of *FAF1/2c* in the *EF* mutant and the *FAF1/2c*‐OE lines in the AC background both led to early flowering (Figures 1, 2, 3). This new function of *FAF* genes in flowering time control in tomato has thus expanded the spectrum of the roles of *FAF* genes in plants.

Plant genomes are mostly composed of non‐coding regions that contain CREs. Although these sequences do not alter protein structures, they could affect the expression levels of genes and the accumulation of proteins. Variations in CREs can lead to drastic changes in gene expression levels and phenotypes. Several recent studies have shown that variations in the promoter regions can alter gene expression in different plants, including rice (Si *et al*., 2016), maize (Tian *et al*., 2019) and tomato (Wu *et al*., 2018). Additionally, an increasing number of studies have shown that mutations in the 3′‐regions downstream of the stop codons can change the expression levels of the genes and lead to abnormal phenotypes in *Arabidopsis* (Wang *et al*., 2007) and rice (Yang *et al*., 2016). In this work, we found that a T‐DNA insertion in the 3′‐UTR of *FAF1/2c* increased the expression of *FAF1/2c* (Figure 2). These results demonstrate that genomic DNA sequences downstream of the stop codon of a gene can regulate gene expression. We also found that plants with the T‐DNA insertion in the 3′‐UTR and plants overexpressing *FAF1/2c* were phenotypically similar. These data are consistent with the hypothesis that the T‐DNA insertion disrupts a crucial CRE in the 3′‐UTR that downregulates the expression of *FAF1/2c* in the *EF* mutant (Figure 2). These observations demonstrate a transcript dosage effect of *FAF1/2c* on flowering time and a regulatory role of the 3′‐UTR from *FAF1/2c* (Figures 1 and 2). Although the essential role of the 3′‐UTR in the expression of *FAF1/2c* was described in this study, the underlying molecular mechanism of how the T‐DNA insertion in the 3′‐UTR leads to the accumulation of the *FAF1/2c* mRNA transcript awaits further study. On the one hand, this may be related to the possibility that the 3′‐UTR is involved in the post‐transcriptional regulation of genes, affecting mRNA stability or translation efficiency (Dassi and Quattrone, 2012). The CREs of the 3′‐UTR have been shown to promote mRNA degradation under specific conditions (Simone and Keene, 2013; Tavares *et al*., 2000; Vlasova and Bohjanen, 2008). In the rice mutant, *upward rolled leaf 1* (*url1*), the CNS2 motif, which is conserved in the 3′‐UTR region of the gene, has been found to have a single base substitution (C‐T), resulting in the enhancement of the stability of its mRNA and significantly increased expression of the *URL1* gene in the mutant compared to the wild‐type (Fang *et al*., 2021). Moreover, microRNAs have also been demonstrated to regulate gene expression at the post‐transcriptional level by binding to specific sequences on the 3′‐UTR, mediating mRNA degradation and inhibiting translation (Djuranovic *et al*., 2012). On the other hand, 3′‐UTR can also serve as a regulator of gene transcription. The 3′‐UTR of *SP5G* has been shown to act as an enhancer to promote the expression of *SP5G*, which regulates flowering time in tomato (Soyk *et al*., 2017; Zhang *et al*., 2018b). Thus, we can reasonably infer that the T‐DNA insertion may disrupt the normal function of a CRE of the 3′‐UTR, leading to a change in the stability of the main ORF (mORF) transcript or transactivation of the upstream ORF (uORF). Genome editing of promoters has been applied to the translational control of target gene expression (Hendelman *et al*., 2021; Rodriguez‐Leal *et al*., 2017; Zhang *et al*., 2018a). We applied gene editing technology to edit the promoter region of the *FAF1/2c* gene, resulting in the early flowering of tomato (results not shown). Identification of the CRE in the 3′‐UTR of *FAF1/2c* may provide a new strategy for controlling flowering time by editing the 3′‐UTR and promoter sequences of *FAF1/2c* in tomato.

As a regulator of the Skp1‐cullin‐F box protein (SCF) type of ubiquitin E3 ligases, the COP9 signalosome (CSN) controls proteasome‐mediated protein degradation (Schwechheimer and Deng, 2001; Zhou *et al*., 2001). Interestingly, differential gene expression or post‐translational modification may also affect CSN activities because CSN is known to be phosphorylated (Bech‐Otschir *et al*., 2002). CSN5 is a subunit of the CSN complex that contains the metalloprotease catalytic core (Cope *et al*., 2002; Verma *et al*., 2002). The level of CSN5 has been linked to cell proliferation status in animals and humans (Tomoda *et al*., 2005; Yoshida *et al*., 2013). *CSN5* plays an important role in photomorphogenesis and plant development (Jin *et al*., 2014, 2018; Wei *et al*., 2008; Wei and Deng, 1999). There are two *CSN5* genes in tomato, *SlCSN5A* and *SlCSN5B*. SlCSN5A contributes to the accumulation of anthocyanins by interacting with SlBBX20 to accelerate the degradation of target proteins (Luo *et al*., 2021). To our knowledge, there has been no report to suggest a role of *CSN5B* in flowering time control. Here we report that FAF1/2c interacts with CSN5B and reduces the protein stability of CSN5B (Figures 4 and 6). Notably, *FAF1/2c*‐OE lines and *csn5b*‐CR lines were phenotypically similar in that they flowered early (Figures 3 and 5). These data indicate that FAF1/2c regulates flowering time in tomato by affecting the protein stability of CSN5B and explain why the *CSN5B* knockout lines and the *FAF1/c*‐OE lines were phenotypically similar. However, more biochemical and enzymatic studies are needed to determine the mechanism used by FAF1/2c to affect the stability of CSN5B.

Tomato SFT is the orthologue of *Arabidopsis* FT and serves as the mobile florigen signal that promotes flowering (Lifschitz *et al*., 2006; Shalit *et al*., 2009). *SFT*, *J* and *UF* promote flowering in tomato (Dielen *et al*., 2004; Molinero‐Rosales *et al*., 2004; Szymkowiak and Irish, 2006). It has been shown that florigen may replace the high light dose required for the *UF*‐mediated photoperiodic pathway in tomato and that increasing the expression level of *SFT* in the late‐flowering mutant *uf* results in the *uf* mutant flowering earlier (Lifschitz *et al*., 2006). Previous reports have shown that *J* is located downstream of *UF*, but *UF* does not directly regulate the gene expression of *J* (Quinet *et al*., 2006a,b). The potential regulatory mechanisms in the tomato flowering regulatory pathways remain mostly unknown. In this study, we found significantly enhanced expression levels of *SFT*, *J* and *UF* in *FAF1/2c*‐OE lines and *csn5b*‐CR lines (Figure 7). Thus, the FAF1/2c‐CSN5B module may regulate flowering time by up‐regulating the expression of flower‐promoting factors such as *SFT*, *J* and *UF*. More research work on the molecular mechanism underlying the regulation of *SFT*, *J* and *UF* expression and the promotion of early flowering by the FAF1/2c‐CSN5B module will provide new insight into the mechanism that controls flowering time in plants. With the success of the CRISPR/Cas9 system in plants, the entire flowering pathway, now including *FAF1/2c* and *CSN5B* genes, can be targeted in many crops to test whether customized alleles can benefit crop improvement.

In summary, we identified *FAF1/2c* as a novel regulator of flowering time in tomato. Based on the results of this study, we propose a model for the role of the FAF1/2c‐CSN5B module in regulating the flowering time in tomato (Figure 7c). In this model, a T‐DNA insertion in the 3′‐UTR of *FAF1/2c* in the *EF* mutant enhances the expression of *FAF1/2c*, leading to the accumulation of the FAF1/2c protein. FAF1/2c interacts with CSN5B and reduces its protein stability. Both the accumulation of FAF1/2c protein and the inactivation of CSN5B protein lead to the induction of early flowering by promoting the expression of *SFT*, *J* and *UF*. The *csn5b*‐CR lines could not produce functional CSN5B and display the early flowering phenotype as in the overexpression lines of *FAF1/2c*‐OE plants.

## Materials and methods

### Plant materials and growth conditions

*Solanum lycopersicum* cv. Alisa Crag (AC) was used as the wild‐type (WT) control of tomato. Seeds from AC and transgenic plants were germinated in the soil. Two‐week‐old seedlings were transferred to new soil to grow in a greenhouse at 24 °C and 18 °C in the daytime and nighttime, respectively. The long‐day conditions were set at 16 h of light and 8 h of dark and the short‐day conditions included 8 h of light and 16 h of dark. Flowering time was quantified by counting the number of days and the number of leaves before the emergence of the first inflorescence.

### Tail‐PCR

The *EF* mutant was identified based on its early flowering phenotype from a population of transgenic plants generated using an *Agrobacterium‐*mediated T‐DNA transformation procedure. The genomic regions flanking the T‐DNA insertion were amplified using the genomic DNA of the *EF* mutant, high‐efficiency thermal asymmetric interlaced PCR (Liu and Chen, 2007) and specific primers (Table S1).

### Vector construction

For overexpression experiments, the full‐length cDNAs from *FAF1/2c* and *CSN5B* were amplified and introduced into the binary vector pHellsgate8 using the ClonExpress II One Step Cloning Kit according to the manufacturer manual (Vazyme, Nanjing, China, http://www.vazyme.com/). For gene editing, *FAF1/2c* and *CSN5B* were targeted with two single‐guide RNAs (sgRNAs) each. The sgRNAs were designed using the CRISPR‐P 2.0 website (http://crispr.hzau.edu.cn/CRISPR2/) and were introduced into the CRISPR/Cas9 binary vector as described previously (Ye *et al*., 2017) using specific primers (Table S1).

### RNA isolation and gene expression analysis

Total RNA was extracted from plant samples using Trizol reagent (Invitrogen) following the manufacturer's instructions. cDNA was reverse transcribed from 3 μL of total RNA using the HisScript II 1st Strand cDNA Synthesis Kit (Vazyme, Nanjing, China; http://www.vazyme.com/). Relative gene expression levels were quantified using qRT‐PCR with the SYBR Green SuperMix and specific primers (Table S1). The *Actin* gene (Solyc11g008430) was used as an internal standard. Three technical replicates were performed for each assay. Relative expression was calculated using the 2^−ΔΔCT^ method (Livak and Schmittgen, 2001).

### Yeast two‐hybrid assays

A tomato cDNA library in a yeast activation domain (AD) fusion vector (Luo *et al*., 2013) was constructed from total RNA isolated from AC. The library was screened using the coding sequence (CDS) from *FAF1/2c* as bait. Plasmids from positive clones were sequenced using the T7 and 3′‐AD primers. For verification of the interaction between FAF1/2c and COP9 signalosome subunit 5B (CSN5B), the full‐length CDS of *FAF1/2c* and various truncated CDS fragments of *CSN5B* were amplified and cloned in pGBKT7 and pGADT7 vectors, respectively. The pairs of plasmids were co‐transformed into *Saccharomyces cerevisiae* strain AH109. Transformants were plated on SD/‐Trp‐Leu medium for cell growth and SD/‐Trp‐Leu‐Ade‐His medium to select for protein–protein interactions. Plasmids were constructed using specific primers (Table S1).

### Luciferase reporter analysis

Luciferase (LUC) reporter assays were performed as described previously (Wang *et al*., 2021). A 3‐kb promoter sequence from *FAF1/2c* was used to drive the expression of the LUC coding‐sequence fused to fragments containing various amounts of the 3′‐UTR. The fusion sequences were cloned into the pluc‐35Rluc vector (Zhang *et al*., 2018b). The CDS from *FAF1/2c* was cloned into pGreen II cLUC and the CDS from *CSN5B* was cloned into pGreen II nLUC. The plasmids were constructed using specific primers (Table S1). Tobacco leaf epidermis cells were co‐transformed with these two plasmids using *Agrobacterium*‐mediated infiltration. LUC activities were measured using a live‐cell system.

### Protein pull‐down analysis

The CDSs of *FAF1/2c* and *CSN5B* were cloned into plasmids useful for expressing proteins with glutathione‐*S*‐transferase (GST) and maltose‐binding protein (MBP) fusion proteins. The GST‐FAF1/2c and GST‐CSN5B recombinant fusion proteins were expressed in *Escherichia coli* strain BL21. MBP‐FAF1/2c and MBP‐CSN5B were expressed in *E. coli* strain DE3. Protein expression was induced with 0.3 mM isopropyl‐β‐D‐thiogalactopyranoside (IPTG). The GST‐ and MBP‐fusion proteins were purified with glutathione sepharose and amylose agarose beads, respectively. The pull‐down assay to test for interactions between FAF1/2c and CSN5B was adapted from a previously described protocol (Feng *et al*., 2008).

### Protein extraction and Co‐IP assays

The full‐length *FAF1/2c* and *CSN5B* cDNAs were amplified and cloned into pHellsgate8 and pH7LIC vector for expressing FAF1/2c‐mCherry and CSN5B‐HA fusion proteins. The recombinant vectors were used to co‐express the two proteins tagged with either the mCherry tag or the HA tag in tobacco leaves. Protein extraction and Co‐IP assays were performed as reported previously, with modifications. The precipitated proteins were analysed by SDS‐PAGE and immunoblotting using anti‐mCherry and anti‐HA antibodies. A chemiluminescent substrate (TransGen, Beijing, China) was used as the developing solution for images.

### Statistical analyses

To analyse the flowering time of plants, five individual plants were used to record the flowering time. R software (https://www.r‐project.org/) was used for statistical calculations in the two‐way analysis of variance. The Student's *t*‐test was performed to compare the mean values for each measured parameter.

## Author contributions

D.Z., G.A., K.J., R.H., L.C., J.W., G.L., Z.Y., C.C., X.W., T.W., J.Y., Z.H., Z.Y. and J.Z designed and planned experiments. D.Z., G.A., R.H., K.J., L.C., J.W., G.L., Z.Y. and C.C. performed experiments and collected the data. D.Z., G.A. and J.Z. analysed the data. D.Z., G.A. and J.Z wrote the manuscript. D.Z., G.A., X.W., M.T., T.W., J.Y., Z.H., Z.Y. and J.Z revised the manuscript.

## Conflict of interest statement

The authors declare no competing interests.

## Supporting information


**Figure S1** Fruit yield in *EF* mutant.
**Figure S2** Gene structure and expression pattern of *SlFAF1/2c*.
**Figure S3** Relative expression levels of FAF1/2c and phenotype of FAF1/2c‐OE transgenic lines.
**Figure S4** Phenotype of overexpressing *CSN5B* transgenic plants.
**Figure S5** Roles of *FAF1/2c* and *CSN5B* on the expression of the flowering genes in tomato.

**Table S1** Primers used in this research.
