# Supplementary material for: EARLY FLOWERING is a dominant gain‐of‐function allele of FANTASTIC FOUR 1/2c that promotes early flowering in tomato
Source: Plant Biotechnol J. 2023 Nov 6;22(3):698–711. doi: 10.1111/pbi.14217 (PMC10893951; doi:10.1111/pbi.14217)
Supplement: Supplementary file 1 — Figure S1 Fruit yield in EF mutant. Figure S2 Gene structure and expression pattern of SlFAF1/2c. Figure S3 Relative expression levels of FAF1/2c and phenotype of FAF1/2c‐OE transgenic lines. Figure S4 Phenotype of overexpressing CSN5B transgenic plants. Figure S5 Roles of FAF1/2c and CSN5B on the expression of the flowering genes in tomato. [file PBI-22-698-s002.docx]

**Supplemental Figures and figure legends**


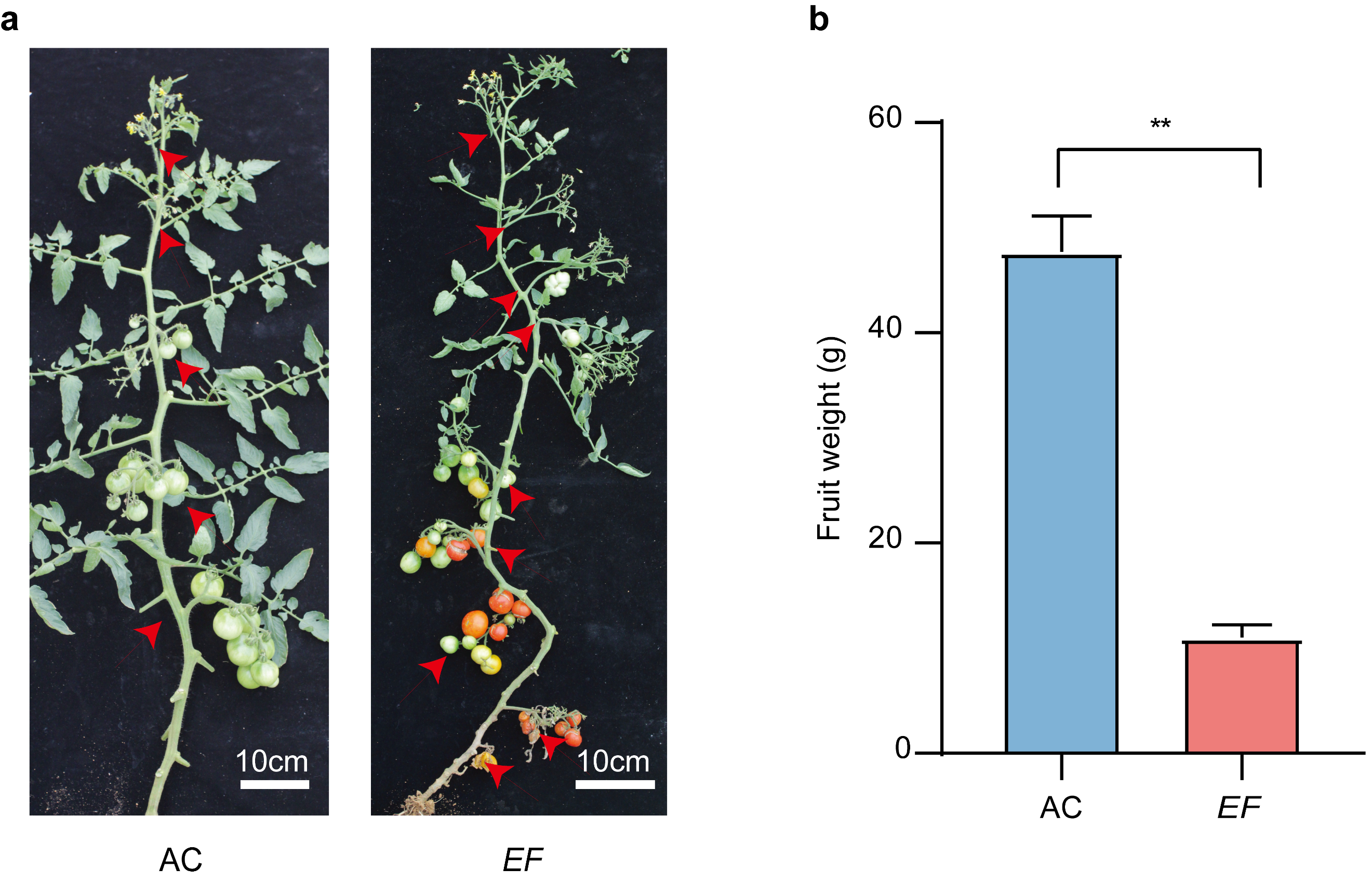


**Fig. S1 Fruit yield in *EF* mutant**

(a) Comparison in fruit setting between AC and *EF* mutant. More fruits were developed in the *EF* mutant than in AC plants. Red arrows indicate the positions of inflorescences. Scale bars, 10 cm. (b) Comparison in fruit weight between AC and *EF* mutant plants. Asterisks indicate statistically signiﬁcant differences between *EF* mutant and AC. **, *P* < 0.01.


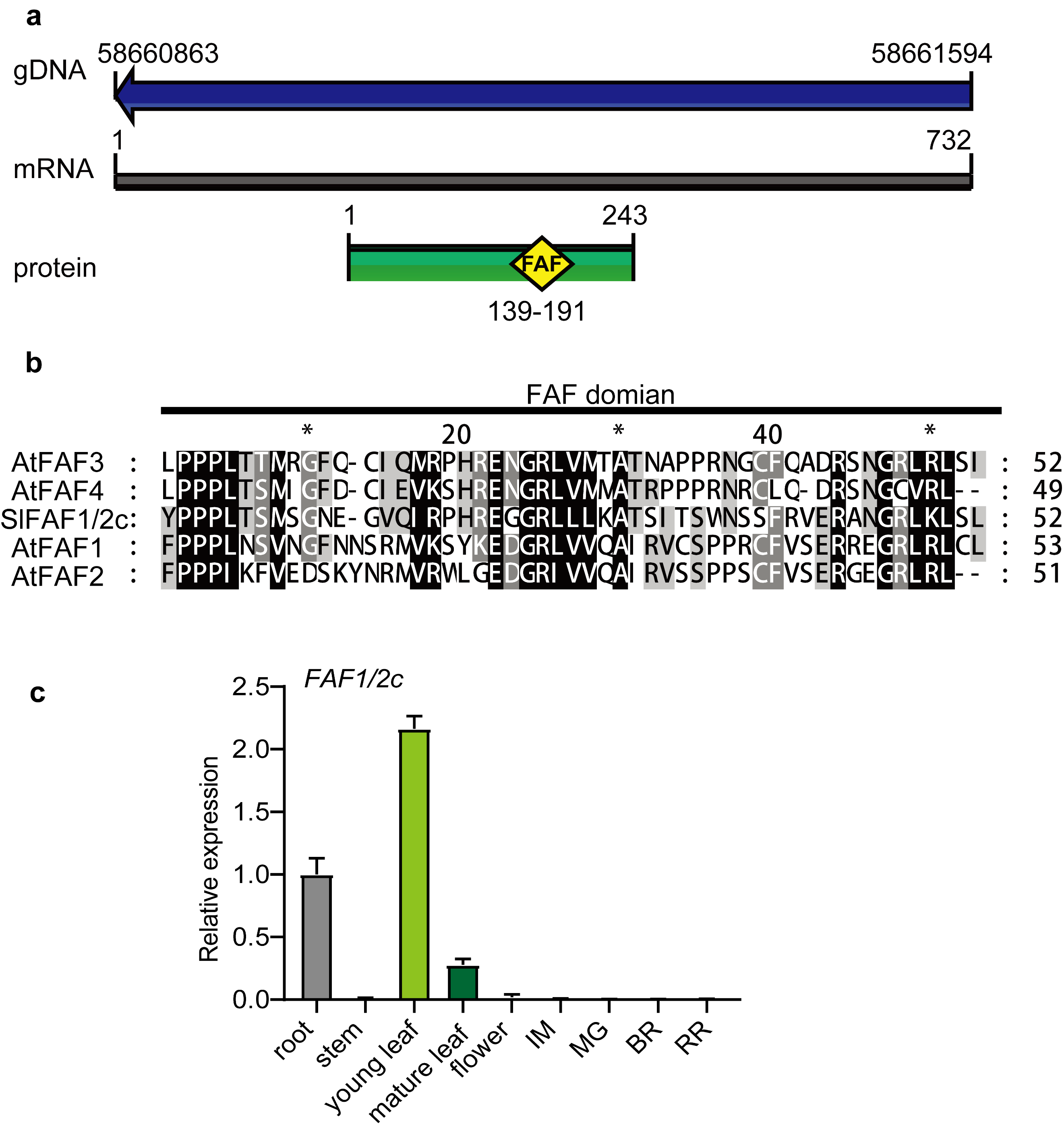


**Fig. S2 Gene structure and expression pattern of *SlFAF1/2c***

(a) Structure of the *FAF1/2c* gene. The gene does not contain any intron. Its mRNA and protein are shown in a black line and a green rectangle, respectively. The characteristic conserved FAF domain of the FAF protein family is indicated as a yellow diamond. (b) Amino acid sequence alignment of the FAF domains of AtFAF1, AtFAF2, AtFAF3, AtFAF4, and SlFAF1/2c. Residues conserved in at least three of the five sequences are shaded grey and residues conserved in all the sequences are shaded black. (c) The relative expression levels of *Sl**FAF1/2c* in different tissues of Ailsa Craig (AC) were measured by qRT-PCR. The expression level in roots was set at 1.0. IM, immature green fruit; MG, mature green fruit; BR, breaker fruit; RR, red ripe fruit. All data are shown as mean values ± SE (n=3).


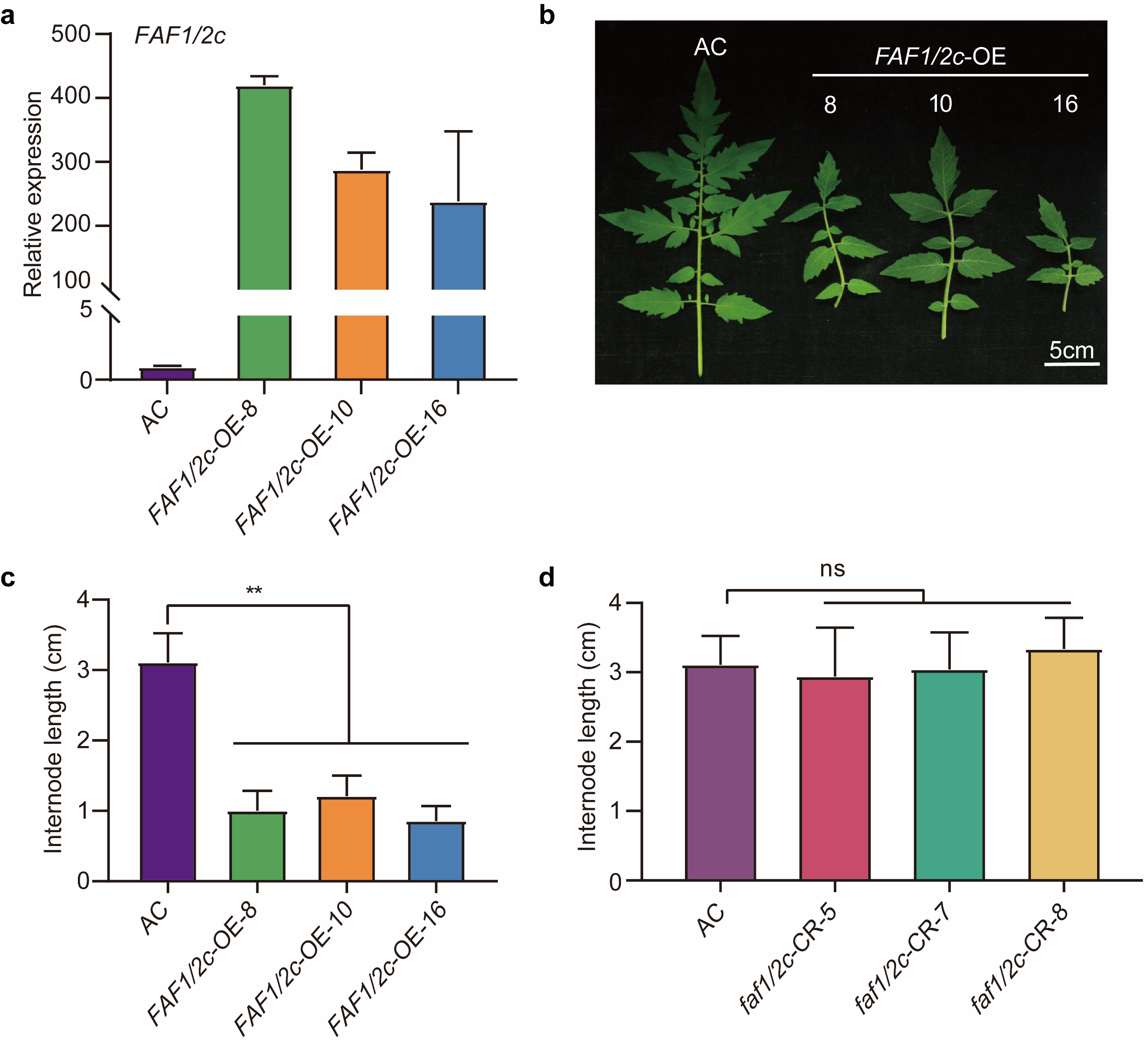


**Fig. S3 Relative expression levels of *FAF1/2c* and phenotype of *FAF1/2c*-OE transgenic lines**

(a) Relative expression levels of the *FAF1/2c* transcript in *FAF1/2c*-OE lines in comparison with that of the control AC plant. The expression level of the *FAF1/2c* transcript in AC was set at 1.0. (b) Leaf phenotype of *FAF1/2c*-OE transgenic lines as compared with that of AC plants. (c) Internode length of *FAF1/2c*-OE transgenic lines as compared with that of AC plants. **, *P* < 0.01. (d) Internode length of *faf1/2c*-CR lines as compared with that of AC plants. ns, no significant difference in statistical analysis.


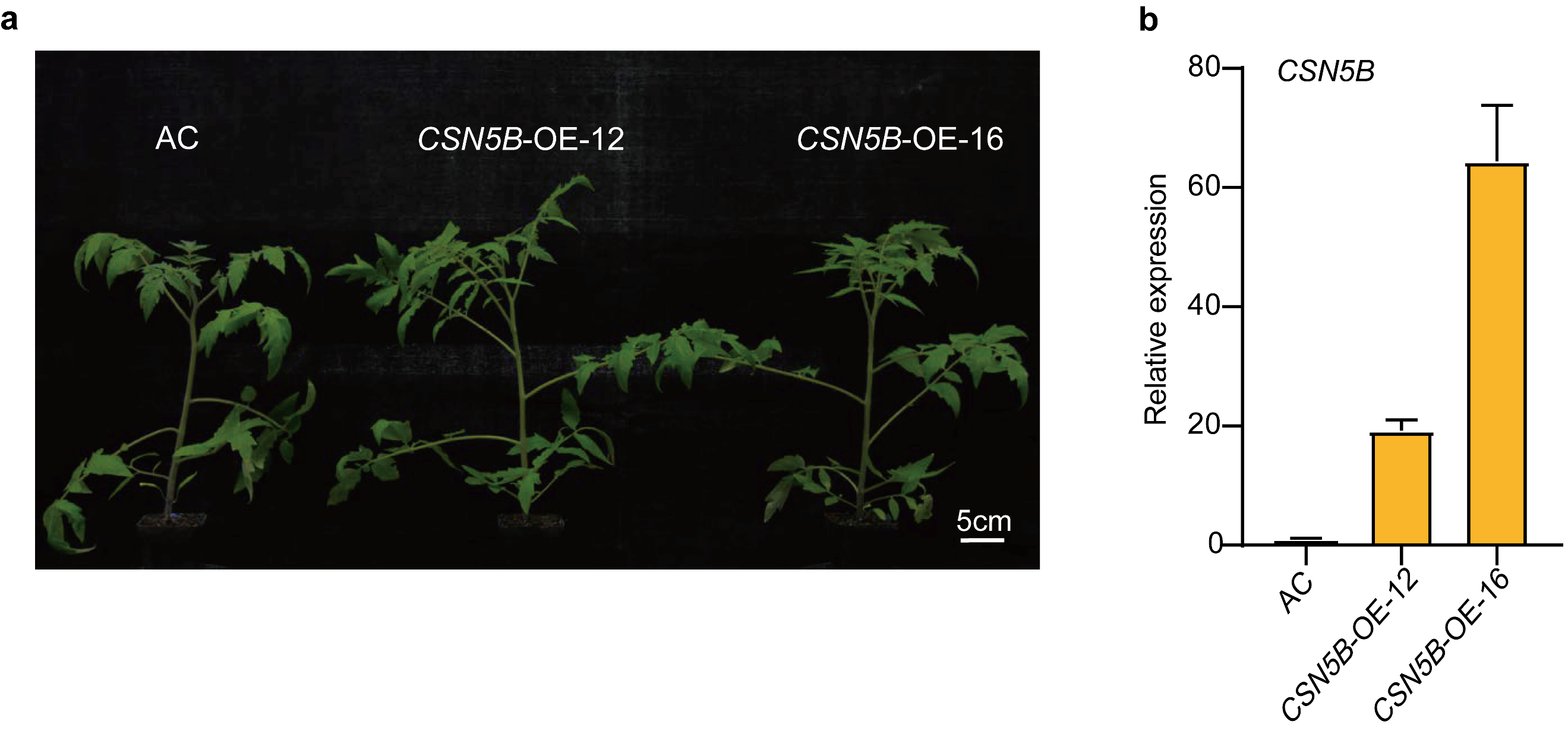


**Fig. S4 Phenotype of overexpressing *CSN5B* transgenic plants**

(a) Phenotype of transgenic plants of *CSN5B*-OE lines (OE-12 and OE-16) as compared with the control plant of AC. Note that no early flowering phenotype was observed in *CSN5B*-OE lines. Scale bars, 5 cm. (b) Relative expression levels of the *CSN5B* transcript in *CSN5B*-OE lines in comparison with that of the control AC plant. The expression level of the *CSN5B* transcript in AC was set at 1.0.


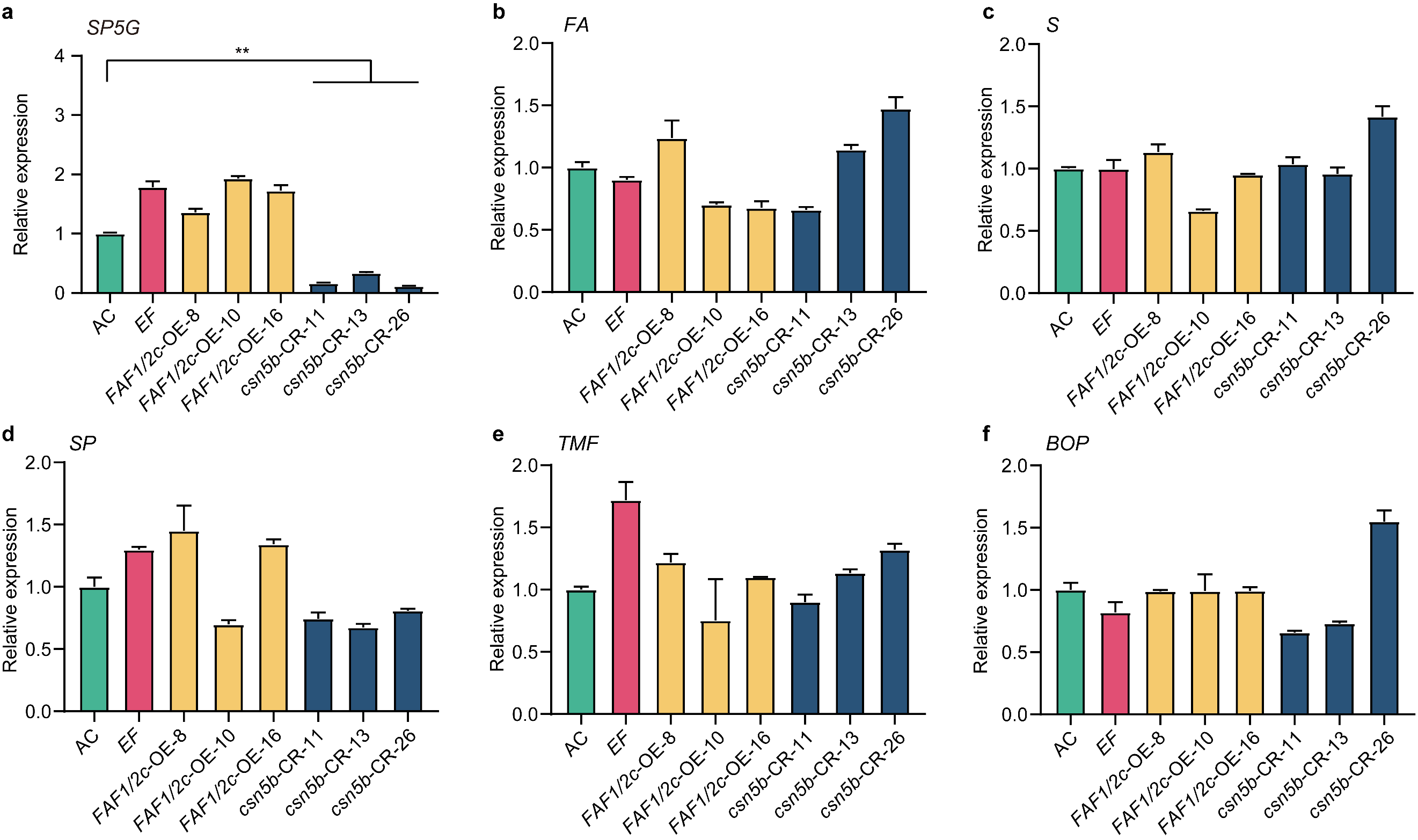


**Fig. S5** Roles of *FAF1/2c* and *CSN5B* on the expression of the flowering genes in tomato.

Relative expression levels of *SP5G* (a), *FA* (b), *S* (c), *SP* (d), *TMF* (e), and *BOP* (f) in the *EF* mutant, *FAF1/2c*-OE, and *csn5b*-CR lines. The expression levels of *SP5G*, *FA*, *S*, *SP*, *TMF*, and *BOP* in the control AC plants were set at 1.0, respectively. **, *P* < 0.01, *t*-test, (n=3).
